# Ginsenoside Rb1 ameliorates Glycemic Disorder in Mice With High Fat Diet-Induced Obesity *via* Regulating Gut Microbiota and Amino Acid Metabolism

**DOI:** 10.3389/fphar.2021.756491

**Published:** 2021-11-24

**Authors:** Xueyuan Yang, Bangjian Dong, Lijun An, Qi Zhang, Yao Chen, Honglin Wang, Ziteng Song

**Affiliations:** ^1^ State Key Laboratory of Medicinal Chemical Biology, College of Pharmacy, Tianjin Key Laboratory of Molecular Drug Research, Nankai University*,* Tianjin, China; ^2^ School of Pharmacy, Shanghai Jiao Tong University, Shanghai, China

**Keywords:** ginsenoside Rb1, insulin sensitivity, gut microbiota, amino acid metabolism, correlation analysis

## Abstract

Accumulating evidences suggested an association between gut microbiome dysbiosis and impaired glycemic control. Ginsenoside Rb1 (Rb1) is a biologically active substance of ginseng, which serves anti-diabetic effects. However, its working mechanism especially interaction with gut microbes remains elusive in detail. In this study, we investigated the impact of Rb1 oral supplementation on high fat diet (HFD) induced obesity mice, and explored its mechanism in regulating blood glucose. The results showed that higher liver weight and lower cecum weight were observed in HFD fed mice, which was maintained by Rb1 administration. In addition, Rb1 ameliorated HFD induced blood lipid abnormality and improved insulin sensitivity. Several mRNA expressions in the liver were measured by quantitative real-time PCR, of which UCP2, Nr1H4, and Fiaf were reversed by Rb1 treatment. 16S rRNA sequencing analysis indicated that Rb1 significantly altered gut microbiota composition and increased the abundance of mucin-degrading bacterium *Akkermansia* spp. compared to HFD mice*.* As suggested via functional prediction, amino acid metabolism was modulated by Rb1 supplementation. Subsequent serum amino acids investigation indicated that several diabetes associated amino acids, like branched-chain amino acids, tryptophan and alanine, were altered in company with Rb1 supplementation. Moreover, correlation analysis firstly implied that the circulation level of alanine was related to *Akkermansia* spp.. In summary, Rb1 supplementation improved HFD induced insulin resistance in mice, and was associated with profound changes in microbial composition and amino acid metabolism.

## Introduction

The prevalence of overweight and obesity is increasing at an alarming rate and is a leading risk factor for diseases such as diabetes, non-alcoholic fatty liver disease (NAFLD), and a plethora of cardiovascular diseases (CVD) (Kusminski, et al., 2016; Yoon et al., 2021). The causes of obesity and overweight are complex and may involve genetic, nutritional, and environmental factors. The major cause is an energy imbalance due to excessive calories consumption and intake of a high fat diet (HFD). The liver, as a 'metabolic workhorse', performs a diverse array of biochemical functions necessary for whole-body metabolic homeostasis, which is related to alterations in glucose, fatty acid, bile acid, insulin resistance, lipoprotein metabolism and inflammation in obese people, and is a major target of clinical anti-type 2 diabetes mellitus (T2DM) drugs like metformin (Foretz, et al., 2019; Yang, et al., 2018).

Nowadays mounting evidences suggest a significant link between gut microbiome and human metabolic health, especially in T2DM (Canfora, et al., 2019; Diez-Echave et al., 2020; Meijnikman, et al., 2018; Petersen, et al., 2017). Obesity is often associated with gut bacterial dysbiosis, with recent evidence revealing a significant difference in the gut microbiota between lean and obese individuals. The gut microbiota mainly enriched in the cecum and proximal colon could metabolize dietary fibers, proteins, and peptides, which escape digestion of host enzyme, suggesting that different abundance in specific genus or species has effect on human health (Koh et al., 2016). An increased ratio of the major phyla Firmicutes/Bacteroidetes and changes in several bacterial species could promote the development of obesity in both dietary and genetic models of obesity in mice (Ley et al., 2005; Turnbaugh et al., 2009). Some bacterial species like *Akkermansia* spp. were considered as probiotics and put into great hopes to be developed as new agents for the treatment of metabolic syndrome (Anhe et al., 2015; Plovier et al., 2017; Barcena et al., 2019). Their effects may be mediated partly through the metabolome, such as short chain fatty acid (SCFA), bile acids, amino acids (AAs), trimethylamine N-oxide, and so on. Among them, AAs are emerging as a new important class of effective molecules in the etiology of obesity and diabetes mellitus. Alternations in blood concentrations of select amino acids and their derivatives, in particular branched-chain amino acids (BCAAs), sulfur amino acids, tyrosine (Tyr), and phenylalanine (Phe), are apparent with insulin resistance, often before the onset of clinically diagnosed T2DM (Agus, et al., 2018; Lynch and Adams, 2014; Nie et al., 2018). BCAAs, including leucine (Leu), isoleucine (Iso), and valine (Val), are essential amino acids synthesized particularly by the regulation of the gut microbiota, playing a critical role in human body weight maintaining and glucose homeostasis (D'Antona et al., 2010). However, the circulating levels of BCAA tend to elevate in obese individuals, and T2DM patients (Karusheva et al., 2019), and high levels of BCAAs in serum are associated with inferior metabolic status and insulin resistance, probably a consequence of the 20% increased consumption of calories over the past 50 years (Newgard, 2012). Therefore, maintaining BCAAs balance is effective and beneficial to the modulation of metabolic syndrome.

Ginseng is an herbal medicine that has been used for improving overall health worldwide (Wong, et al., 2015). It has been considered as the most valuable of all medicinal plant in Asian for its broad health benefits documented in medical book as well as traditional belief (Nahin, et al., 2009; Sánchez et al., 2017). Ginsenosides, representative as Rb1, Rg1, Rg3, Re, and Rd, are the major class of bioactive components in Ginseng (Shergis, et al., 2013). Of the various ginsenosides, Rb1 (PubChem CID: 9898279), a most abundant and bioactive molecule of Ginseng, has been reported various pharmacological activities, such as anti-diabetic effect in many *in vitro* and *in vivo* models (Zhou et al., 2019; Fan et al., 2020). Rb1 is deglycosylated by intestinal bacteria after oral intake, and converted to minor ginsenosides, like compound K. The metabolites generated by intestinal microflora are more permeable and bioactive (Shen et al., 2013). Up to date, the elaborate working mechanism of Rb1 on glucose control has not been clearly understood, which is related to gut microbiota due to the degradation in gastrointestinal tract. Rb1 was reported to modulate the gut microbial composition, but the correlation between the altered gut microbiota, related metabolites and glucose lowering effects remains unclear in detail (Bai et al., 2021). Based on this, we endeavored to evaluate the metabolic effects of the Rb1 and explore its regulatory role on gut microbiome and amino acid metabolism in HFD induced obesity and diabetes in mice.

## Materials and Methods

### Chemicals and Reagents

**FIGURE 1:**
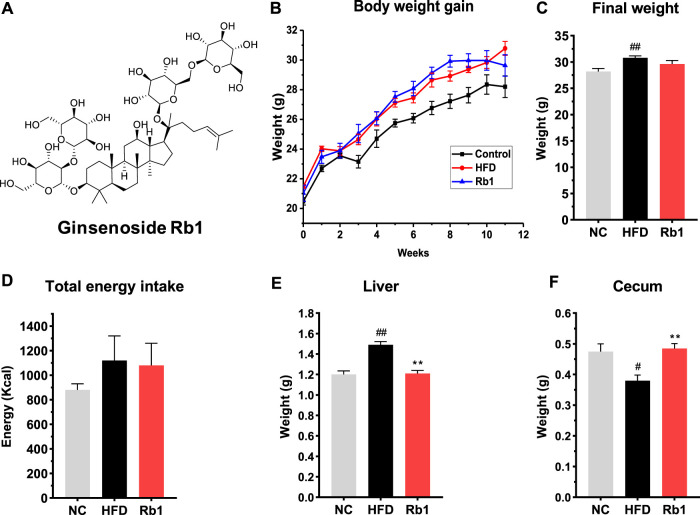
The effects of Rb1 supplementation on body composition. **(A)** structure of ginsenoside Rb1; **(B)** Weight gain assessed weekly; **(C)** Final body weight; **(D)** Total energy intake; **(E)** Liver weight; **(F)** Cecum weight. The data are expressed as the mean ± SEM; n = 6; one-way analysis of variance with Tukey’s post-hoc test. ^#^ *p* < 0.05, ^##^ *p* < 0.01, for NC vs HFD; ^*^ *p* < 0.05, ^**^ *p* < 0.01 for Rb1 vs HFD.

Ginsenoside Rb1 (>98% purity) was purchased from Desite Co., Ltd. Chengdu. The structure of Rb1 was shown in Figure 1A. The nuclear magnetic resonance (NMR) spectra obtained for Rb1 were shown in Supplementary Figures S1, 2. The HFD was purchased from Trophic Animal Feed High-Tech (Catalog number: TP 23300). Methanol and acetonitrile were provided by Sigma-Aldrich (St. Louis, MO, United States). Acetone and other analytical chemical solvents were obtained from Tianjin Chemical Factory (Tianjin, China).

### Animals

Six-week-old C57BL/6J specific pathogen-free (SPF) male mice were raised in 5–6 per cage in rooms with constant temperature (25 ± 2°C), humidity (50 ± 20%), 12 h dark-light cycle, and free access to normal chow diet and water. After adaption for a week, mice (n = 18) were randomly separated into three groups, NC group and HFD group were fed with normal chow diet and high fat diet food, respectively, while Rb1 group received oral doses of ginsenoside Rb1 (200 mg/kg/day) and high fat diet food simultaneously. The food ingredients are listed in Supplementary Table S1. Mice were assessed weekly for body weight and food intake index. After 12 weeks of feeding, mice were euthanized, blood and faeces were collected at the end of experiments. All samples were collected and stored at −80°C until analysis. All procedures were performed in accordance with the guidelines of the Animal Care and Use Committee of Nankai University, Tianjin, China (2006–0009), and conformed to the Guide for the Care and Use of Laboratory Animals publish Ted by the National Institutes of Health (NIH Publications No. 8023, revised 1978). The Committee approval number is NO. SYXK (JIN) 2019-0001.

### Serum Sample Preparation

At the end of animal experiment, all mice were euthanatized with diethyl ether to collect blood. The blood samples were placed at room temperature for 15 min, and then were centrifuged at 3,000 rpm for 15 min to obtain serum. All serum samples were stored at −80°C for further experiments.

### Biochemical Indexes Analysis

Total triglyceride (TG), total cholesterol (TC), low-density lipid cholesterol (LDL-C), high-density lipid cholesterol (HDL-C), and free fatty acids (FFA) in serum together with TG in liver were measured by Elisa kit (Nanjing Jiancheng Bioengineering Institute). All protocols were conducted according to manufacturer recommendations.

### Glucose Homeostasis Analysis

At week 10, mice were fasted for 12 h and glucose tolerance test (GTT) was performed after an intraperitoneal injection of glucose (1 g/kg body weight). Blood was collected at tail vein and the concentration was measured with an Accu-Check glucometer (Roche). Blood glucose levels were tested before (0 min) and after (15, 30, 60, 90 and 120 min) for glycaemia determination. At the end of week 11, animals were fasted for 4 h, and an insulin tolerance test (ITT) was performed after an intraperitoneal injection of insulin (0.7 UI/kg). Blood glucose concentrations were measured before (0 min) and after (15, 30, 60, 90 and 120 min) insulin injection.

### Quantitative Real-Time PCR

Total RNA was extracted from liver. RNA was used for cDNA synthesis using a reverse transcription PCR kit (TransGen Biotech). Real-time qPCR was performed using Tip Green qPCR SuperMix (TransGen Biotech) with 1:10 diluted cDNA product from the transcription product. GAPDH (Glyceraldehyde-3-phosphate dehydrogenase) was used as housekeeping gene. Data was calculated according to 2^ΔΔCt^ method. Primer sequence for targeted mouse gene was available in Table 1.

**TABLE 1:** Primer sequences used for real-time PCR amplification.

**Gene**	**Forward sequence**	**Reverse sequence**
PPARα	CGA​CCT​GAA​AGA​TTC​GGA​AA	GGC​CTT​GAC​CTT​GTT​CAT​GT
PPARγ	CAG​GCC​TCA​TGA​AGA​ACC​TT	GCA​TCC​TTC​ACA​AGC​ATG​AA
UCP2	CTA​CTG​TCG​AGG​AGA​TCG​AG	GCA​GCA​GTT​TGG​GTT​GTT​TC
Nr1h4	CTG​AGA​CTG​GGT​ACC​AGG​G	CCA​TTC​GCG​GCT​TCT​TTG​TC
Fiaf	CAA​TGC​CAA​ATT​GCT​CCA​ATT	TGGCCGTGGGCTCAGT
Fgfr4	GTA​CCC​TCG​GAC​CGC​GGC​ACA​TAC	GCC​GAA​GCT​GCT​GCC​GTT​GAT​G
GAPDH	CAA​GGT​CAT​CCA​TGA​CAA​CTT​TG	GGC​CAT​CCA​CAG​TCT​TCT​GG

### Gut Microbiota Analysis

Faeces DNA was extracted using PowerSoil DNA Isolation Kit (MoBio Laboratories, Carlsbad, CA) following the manual. Purity and quality of the faeces genomic DNA were checked on 0.8% agarose gels. The V3-4 hypervariable regions of bacterial 16S rRNA gene were amplified with the primers 338F (ACT​CCT​ACG​GGA​GGC​AGC​AG) and 806R (GGACTACHVGGGTWTCTAAT). The PCR products were purified using a QIAquick Gel Extraction Kit (QIAGEN, Germany), quantified using Real Time PCR, and sequenced at Allwegene Company, Beijing. Deep sequencing was performed on Miseq platform at Allwegene Company (Beijing). After the run, image analysis, base calling and error estimation were performed using Illumina Analysis Pipeline Version 2.6. The raw data were first screened, and sequences were removed from consideration if they were shorter than 200 bp, had a low-quality score (≤20), contained ambiguous bases or did not exactly match to primer sequences and barcode tags. Qualified reads were separated using the sample-specific barcode sequences and trimmed with Illumina Analysis Pipeline Version 2.6. And then the dataset was analyzed using QIIME. The sequences were clustered into operational taxonomic units (OTUs) at a similarity level of 97% (Edgar, 2013), to generate rarefaction curves and to calculate the richness and diversity indices. The Ribosomal database Project (RDP) Classifier tool was used to classify all sequences into different taxonomic groups (Cole et al., 2008). To examine the similarity between different samples, clustering analyses and principal component analysis (PCA) were used based on the OTU information from each sample using R (Wang et al., 2012). The evolution distances between microbial communities from each sample were calculated using the tayc coefficient and represented as an Unweighted Pair Group Method with Arithmetic Mean (UPGMA) clustering tree describing the dissimilarity (1-similarity) between multiple samples (Jiang et al., 2013). A Newick-formatted tree file was generated through this analysis. To compare the membership and structure of communities in different samples, heat maps were generated with the top 20 OTUs using Mothur (Jami, et al., 2013).

### Phylogenetic Investigation of Communities by Reconstruction of Unobserved States (PICRUSt) Analysis

PICRUSt was used to performed the function profiling of microbial communities’ prediction (Langille et al., 2013). OTU table was normalized by 16S copy number predictions so that OTU abundance more accurately reflected the true abundances of the underlying organisms. Predicted functional genes were categorized into Clusters of Orthologous Groups (COG) and into Kyoto Encyclopedia of Genes and Genome (KEGG).

### Short Chain Fatty Acids (SCFAs)

Fecal samples (100 mg) were supplemented with 10 μL of internal standards and 500 μL of methanol, and then extracted by vortexing for 1 min. After centrifuged at 12,000 rpm and 4°C for 10 min, the supernatant was detected using a GC–MS system (Agilent Technologies, Santa Clara, CA), equipped with a polar HP-FFAP capillary column. Injection volume was 2 μL and helium was used as a carrier gas at a constant flow rate of 2 ml/min through the column. The initial oven temperature was kept at 80°C for 1°min, raised to 170°C at a rate of 10°C/min, to 220°C at a rate of 20°C/min, and finally kept 220°C for 3 min. SCFAs standards were mixtures of acetic acid, propionic acid, butyrate, isobutyric acid, valeric acid, and isovaleric acid.

### Serum Amino Acids

10 μL serum was mixed with 20 μL methanol, vortexed for 1 min, and then centrifuged at 4°C for 15 min. Supernatants were collected, and 2.5 μL supernatant or amino acids mixture standards solution was combined with 2.5 μL orthophthalaldehyde (OPA). High performance liquid chromatography (HPLC) Agilent 1,100 series system equipped with a diode array detector was used to analyze 5 μL mixed solution, fluorescence is monitored at excitation and emission wavelengths of 340 and 460 nm. The separation was performed on a Kromasil C18 reverse-phase column (250 mm × 4 mm, 5 μm), protected with an Ultrasep C18 guard column, which was eluted by a binary mobile phase consisting of water containing 0.1% formic acid (A) and acetonitrile (B) with a gradient. The levels of Val, Leu, Iso, histidine (His), serine (Ser), arginine (Arg), glycine (Gly), Tyr, alanine (Ala), phenylalanine (Phe), lysine (Lys), and tryptophan (Trp) were tested, and the chromatogram of amino acids were shown in Supplementary Figures S3, 4.

### Serum Indolepropionic Acid (IPA) and Indoxyl Sulfate (IS)

IPA and IS standard were mixed in 20 μL with 60 μL methanol, and 10 μL serum was mixed with 30 μL methanol. The liquid was vortexed for 1 min, and supernatants were then dried in a SpeedVac. The sediment was resuspended in 50% methanol-water and then underwent HPLC separation on a Kromasil C18 reverse-phase column (250 mm × 4 mm, 5 μm). Water with 0.1% formic acid and acetonitrile were used as mobile phases A and B. The fluorescence was monitored at excitation and emission wavelengths of 280 and 370 nm. The chromatogram of IPA and IAA were shown in Supplementary Figures S5, 6.

### Statistical Analysis

The data were expressed as the mean ± SEM. One-way analysis of variance (ANOVA) followed by Tukey’s post-hoc test was applied to assess intergroup variations in all other cases except gut microbiota results. One-way analysis of variance (ANOVA) followed by kruskal-wallis post-hoc test was applied to assess gut microbiota results. Spearman’s rank test was applied to assess the correlation results. The statistical analysis was performed using GraphPad (GraphPad Prism, United States). A *p*-value of <0.05 indicates a significant difference.

## Results

### Rb1 Exhibits Protective Effects in HFD-Fed Mice

To investigate the protective effect of Rb1 on glucose control, we established a model of HFD-induced mice by raising C57BL/6J with high fat diet feeding for 12 weeks. Rb1 supplementation to HFD mice did not change body weight and energy intake when compared to HFD group (Figures 1B–D). Compared to NC group, liver weight was significantly higher while cecum weight was lower in mice fed with HFD. Significant lower liver weight and higher cecum weight were observed in mice fed with Rb1 as against HFD group (Figures 1E,F).

**FIGURE 2:**
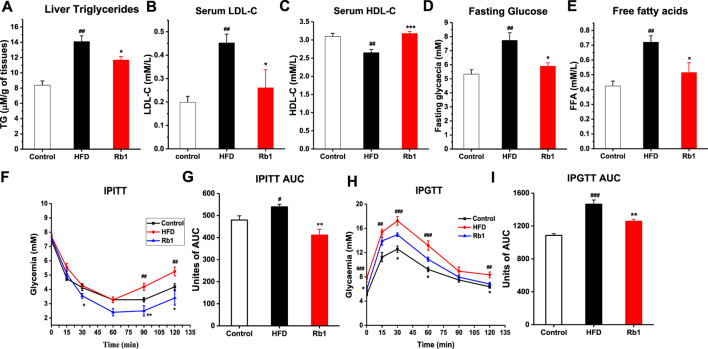
The effects of Rb1 supplementation on lipid profiles and glucose homeostasis. **(A)** Liver triglycerides; **(B)** Low-density lipoprotein cholesterol; **(C)** High-density lipoprotein cholesterol in serum. **(D)** Fasting glucose; **(E)** Free fatty acids in serum. **(F)** Mice for an insulin tolerance test were 6 h fasted and IPITT was performed after an intraperitoneal injection of insulin (0.7 UI/kg body weight). **(G)** Area under the curve for insulin tolerance test. **(H)** Animals were fasted overnight (12 h) and an oral glucose tolerance test IPGTT was performed after gavage with glucose (1 g/kg body weight). **(I)** Area under the curve for IPGTTs. The data are expressed as the mean ± SEM; n = 6; one-way analysis of variance with Tukey’s post-hoc test. ^#^ *p* < 0.05, ^##^ *p* < 0.01, and ^###^ *p* < 0.001 for NC vs HFD; ^*^ *p* < 0.05, ^**^ *p* < 0.01, and ^***^ *p* < 0.001 for Rb1 vs HFD.

Contrasted with NC group, impaired glucose control and insulin resistance as well as higher liver TG, serum LDL-c, HDL-c, fasting glucose and free fatty acids were also observed in mice fed with HFD (Figure 2). In agreement with the observed reduction of liver weight, Rb1 supplementation reduced hepatic triglycerides accumulation (Figure 2A). However, Rb1 did not reverse HFD induced elevated levels of cholesterol and triglycerides in serum (Supplementary Figure S7). Moreover, Rb1-treated mice reduced serum fasting glucose, LDL-c and free fatty acid but increased HDL-c compared to HFD group (Figures 2B–E). In addition, significantly improved of glucose homeostasis and insulin sensitivity were observed by our intraperitoneal insulin tolerance test (IPITT) and intraperitoneal glucose tolerance test (IPGTT) data (Figures 2F–I).

### Rb1 Administration Alters the mRNA Expressions in the Liver

**FIGURE 3:**
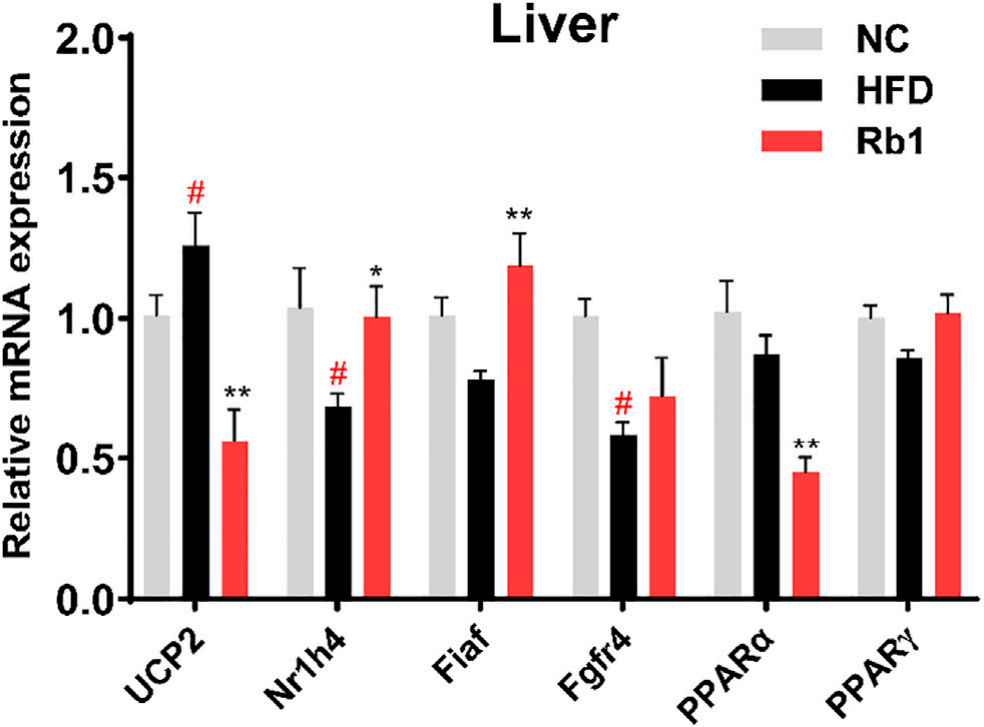
mRNA expression of genes involved in liver were detected by RT-qPCR. Relative expression was calculated using the 2^ΔΔCT^ method with chow mice as the group of reference and GADPH as reference gene. The data are expressed as the mean ± SEM; n = 6; one-way analysis of variance with Tukey’s post-hoc test. ^#^ *p* < 0.05, ^##^ *p* < 0.01, for NC vs HFD; ^*^ *p* < 0.05, ^**^ *p* < 0.01 for Rb1 vs HFD.

Considering the role of liver in the development of obesity, expression levels of hepatic genes involved in glucose metabolism were evaluated. Rb1 treatment significantly down-regulated the uncoupling protein 2 (UCP2) expression in the liver to improve insulin sensitivity (Diano and Horvath, 2012; Kim, et al., 2019). Nuclear receptor subfamily one group H member 4 (Nr1h4) regulates amino acids catabolism and detoxification of ammonium by ureagenesis and glutamine synthesis (Massafra et al., 2017). In addition, the farnesoid X receptor (FXR, encoded by Nr1h4) would regulate the homeostasis of BAs, lipids and glucose, which has been reported to have important role in obesity and hypercholesterolemia (Watanabe et al., 2006). Our analysis revealed that the mRNA expression of Nr1h4 is significantly upregulated by Rb1 (Figure 3). Furthermore, the expression of Fiaf, a circulating inhibitor of lipoprotein lipase, was significantly increased after Rb1 treatment, which was accorded with the decreased liver weight (Bäckhed et al., 2004). Activators of both PPARα and PPARγ have been shown to improve insulin sensitivity and normalize impaired glucose tolerance in both humans and rodent models of insulin resistance (Francque et al., 2021). Rb1 increased the PPARγ expression compared to HFD though did not show significance. In liver, PPARα regulates genes involved in lipid and lipoprotein metabolism (Pawlak, et al., 2015). However, the administration of Rb1 reduced the expression of PPARα, which need to be explored further.

### Effect of Rb1 on Gut Microbiota

**FIGURE 4:**
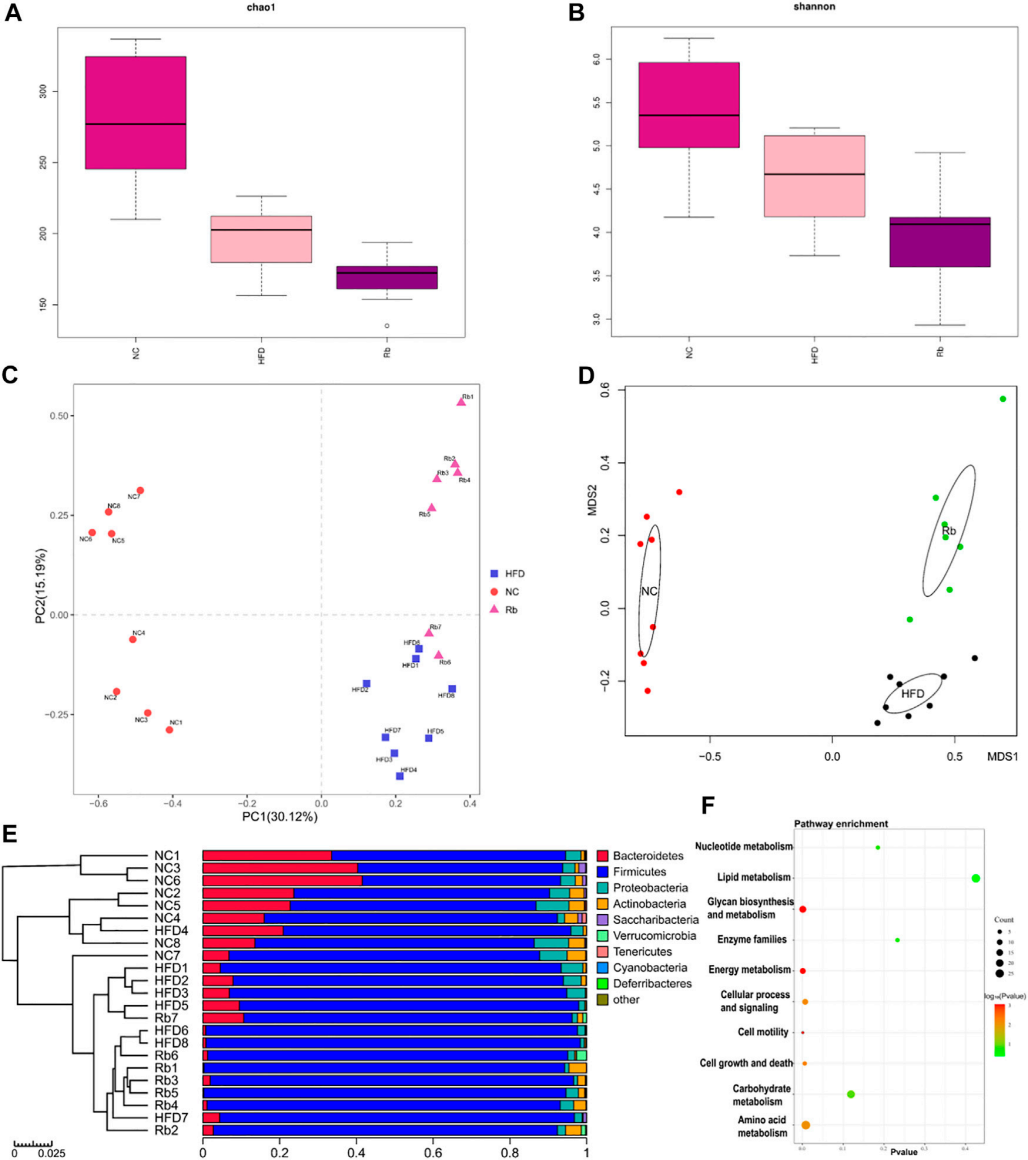
16S rRNA analysis. Faeces were harvested at the end of all experiments, gut microbiota analysis was performed. Alpha diversity analysis of **(A)** chao1 index; and **(B)** Shannon index; Beta diversity analysis of **(C)** Principal component analysis (PCA); and **(D)** Non-metric multidimensional scaling (NMDS) plots. **(E)** Relative abundance of different classes. **(F)** PICRUSt function prediction of the different abundance of gut microbiota in NC, HFD, and Rb1 group (Welch’s *t* test, two-sided).

Previous study demonstrated the synergistic effect of polysaccharides and Rb1 on diabetic model, owing to poor bioavailability of Rb1, the glucose control mechanism related to gut microbes was considered (Li et al., 2018). High-quality gut microbiota sequences were obtained from all the fecal samples of mice by 16S rRNA gene sequencing analysis (Supplementary Figure S8). Microbial *α*-diversity analysis showed that Rb1 did not recover chao1 index and shannon index of HFD-induced mice (Figures 4A,B). Beta diversity analysis using PCA and nonmetric multidimensional scaling (NMDS) exhibited distinct clusters of NC, HFD, and Rb1 groups (Figures 4C,D), which implied that Rb1 treatment affected partial microbiota.

**FIGURE 5:**
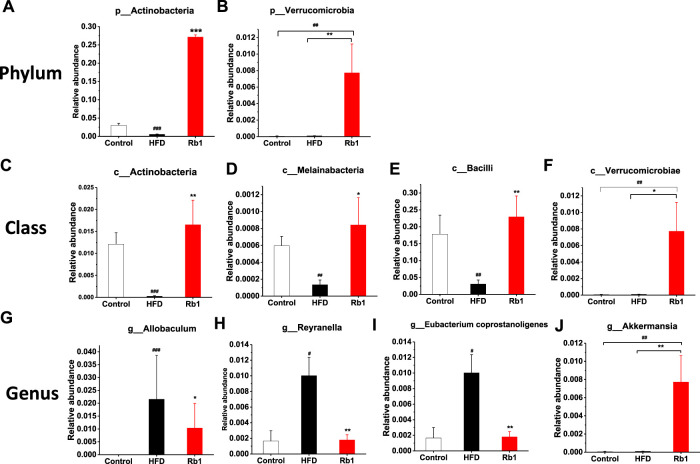
Relative abundance of bacteria in phylum **(A,B)**, class **(C–F)**, and genus **(G–J)** are altered by ginsenoside Rb1. The data are expressed as the mean ± SEM; n = 6; one-way analysis of variance with kruskal-wallis post-hoc test. ^#^ *p* < 0.05, ^##^ *p* < 0.01, and ^###^ *p* < 0.001 for NC vs HFD; ^*^ *p* < 0.05, ^**^ *p* < 0.01, and ^***^ *p* < 0.001 for Rb1 vs HFD.

To examine the changes in greater detail, we next estimated differences using the perspective of cluster analysis. The relative abundance at the phylum, class, order and genus levels were shown in Figure 4E and Supplementary Figures 9A–C. Significant Rb1 induced changes were found for two phyla (*Actinobacteria* and *Verrucomicrobia*), four classes (*Actinobacteria*, *Melainabacteria*, *Verrumcomicrobiae*, and *Bacilli*), and four genera (*Allobaculum* spp. *Reyranella* spp. *Eubacertium coprostanoligenes*, and *Akkermansia* spp.). The relative abundance of phylum *Actinobacteria* was significantly lower in HFD group compared with NC group. Rb1 significantly enriched the abundance of phyla *Verrucomicrobia* and *Actinobacteria*. (Figures 5A,B). The abundance of three classes, *Actinobacteria*, *Melainabacteria*, and *Bacilli* was lowered in HFD group compared with NC group, while Rb1 supplementation significantly reversed this trend (Figures 5C–E). In addition, we observed that the *Verrumcomicrobiae* was markedly enriched in Rb1 group in comparison with both NC and HFD group (Figure 5F). The abundance of three genera, *Allobaculum* spp. *Reyranella* spp. and *Eubacertium coprostanoligenes*, was higher in HFD group compared to NC group, which was significantly reduced after Rb1 treatment (Figures 5G–I). Interestingly, *Akkermansia* spp. known as a probiotic to ameliorate obesity and immune response, was markedly increased by Rb1 (Figure 5J). To reveal the involvement of SCFAs, the main metabolites of gut microbiota, in the Rb1 treatment on HFD-induced mice, six SCFAs in serum were measured. All of the SCFAs were significantly decreased in the HFD-fed mice compared with NC group, which were not recovered by Rb1 supplementation (Supplementary Figure S10).

For the purpose of better understanding Rb1-mediated the gut microbiota changes, we performed the functional profiling of microbial communities’ prediction by PICRUSt. As shown in Figure 4F, the results revealed that the predicted KEGG pathways mainly enriched in amino acid metabolism, glycan biosynthesis and metabolism, and energy metabolism.

### Influence of Rb1 Administration on Amino Acid Metabolism

**FIGURE 6:**
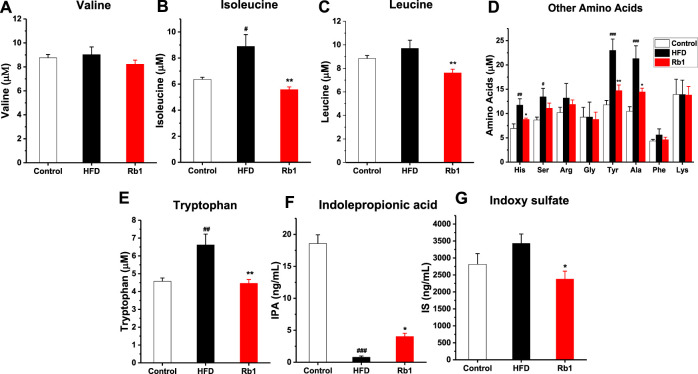
The effects of Rb1 supplementation on amino acids metabolism. **(A)** valine (Val) **(B)** isoleucine (Iso) **(C)** leucine (Leu) **(D)** Other amino acids, histidine (His); serine (Ser); arginine (Arg); glycine (Gly); tyrosine (Tyr); alanine (Ala); phenylalanine (Phe); lysine (Lys) **(E)** tryptophan (Trp) **(F)** indolepropionic acid (IPA) **(G)** indoxyl sulfate (IS) in serum. The data are expressed as the mean ± SEM; n = 6; one-way analysis of variance with Tukey’s post hoc test. ^#^ *p* < 0.05, ^##^ *p* < 0.01, and ^###^ *p* < 0.001 for NC vs HFD; ^*^ *p* < 0.05, ^**^ *p* < 0.01 for Rb1 vs HFD.

Amino acid metabolism, enriched in the predicted KEGG pathways by PICRUSt analysis, plays a vital role in glucose homeostasis, where most of the metabolites then enter into the citric acid cycle to synthesize glucose (Holecek, 2020). Increasing evidences suggested that amino acids are emerging as a new class of effective molecules in the etiology of obesity and diabetes mellitus (Hasanpour, et al., 2020). Among all amino acid analyzed, in contrast to NC group, levels of Iso, His, Ser, Tyr, Ala, and Trp in serum were significantly higher in HFD-fed rats. Meanwhile, Rb1 supplementation decreased levels of Iso, His, Leu, Tyr, Ala, and Trp (Figures 6A–E).

The metabolism of the aromatic amino acid Trp results in numerous effects on immune homeostasis, epithelia function, and gut motility. In addition, as the metabolites of Trp-like indoleacetic acid (IAA), IPA and IS are known to affect intestinal permeability and host immunity (Venkatesh et al., 2014; Huang et al., 2020). The results indicated that tryptophan microbial metabolites IPA was significantly lower in HFD group compared to NC group, and Rb1 supplementation markedly increased the level of IPA and decreased the level of IS (Figures 6F,G).

### Correlation Between Amino Acids and the Gut Microbiota

**FIGURE 7:**
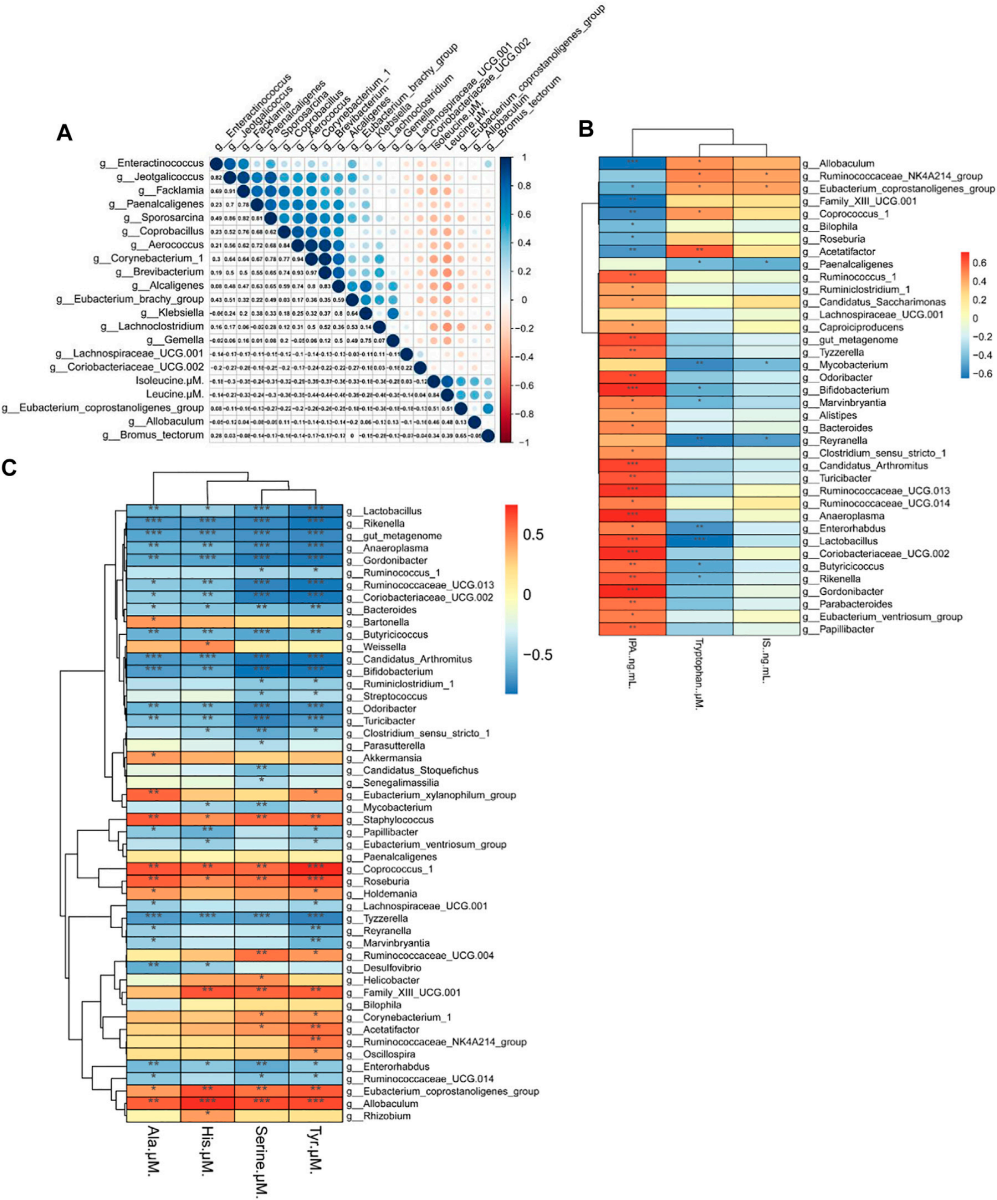
Heat map of the correlation between the gut microbiota and biochemical indexes in mice. **(A)** Correlation between the gut microbiota and Iso (isoleucine), Leu (leucine). **(B)** Correlation of the gut microbiota with Trp (tryptophan), IPA (indolepropionic acid) and IS (indoxyl sulfate) **(C)** Correlation between the gut microbiota and His (histidine), Ala (alanine), Ser (serine), and Tyr (tyrosine). Spearman’s rank test. Significance among different correlation coefficients described as ^*^ *p* < 0.05, ^**^ *p* < 0.01, and ^**^ *p* < 0.001.

BCAAs in particular, are strongly associated with insulin resistance (Zeng et al., 2020). The Spearman’s correlation test was applied to investigate the correlation between amino acids and gut microbiota. As depicted in Figure 7A, 19 genera showed strong correlation with Iso and Leu levels in serum, four genera of which were positively correlated and 15 genera were negatively correlated. Among the 19 genera, *Eubacterium coprostanoligenes* was significantly correlated with Leu and Iso simultaneously.

We also performed Spearman’s correlation analysis of gut microbiota with Trp, IPA and IS. As seen in Figure 7B, 38 genera were correlated with Trp level and its metabolites, and we found that most genera that are negatively correlated with Trp are also positively correlated with IPA. Among 38 genera, *Allobaculum* spp. and *Reyranella* spp. were significantly altered by HFD and Rb1 treatment.

As shown in Figure 7C, 50 genera were correlated with the levels of Tyr, Ser, His, and Ala in serum. *Allobaculum* spp. was positively correlated with Tyr, Ser, and His levels in serum, and *Reyranella* spp. was negatively correlated with Tyr and Ala. Interestingly, we found that Ala was the only amino acid correlated with *Akkermansia* spp., and the relative abundance of *Akkermansia* spp., was significantly increased in Rb1-treated mice. Thus, Rb1 might increase insulin sensitivity by modulating *Eubacterium coprostanoligenes*, *Allobaculum* spp., *Reyranella* spp. and *Akkermansia* spp. to improve the metabolism of amino acids in mice. Moreover, the glucose-alanine cycle necessitates an examination of its quantitative contribution to overall glucose balance, which suggested that Rb1 could regulate homeostasis by normalizing the metabolism of alanine in obese mice (Felig, 1973).

## Discussion

Gut microbiota dysbiosis is closely related to the metabolic diseases such as obesity, inflammatory bowel disease, and cardiovascular disease. As the producers or metabolizers, intestinal bacteria composition will cause the occurrence of metabolic disorders by altering the production of intestinal metabolites, including SCFAs, BAs, BCAAs, and other compounds. Deglycosylation of ginsenosides, mediated by the gut microbiota, has been proposed to play an important role in pharmacological effects. Although the anti-diabetic effect of Rb1 has been documented in several studies, the effect on gut microbiota and its metabolites had not been investigated in detail. In current study, compared with HFD group, Rb1 treatment slightly reduced the body weight but exhibited less trend of body weight gain, which implied that Rb1 would significantly control HFD-induced weight gain after longer administration or higher dose as described for previous report (Zhou et al., 2019). Liver weight is regarded as an intermediate position in the network of correlations that are strongly associated with insulin resistance, and cecum weight is connected with tropic effects of intestinal epithelium cells. Our study first showed that Rb1 prevented liver weight gain and cecum weight loss in the HFD group in an independent manner of the energy intake. In current study, HFD induced the decrease of cecum weight, which is consistent with previous researches (Chen et al., 2019; Berger et al., 2021). The byproducts of lipid metabolism in a HFD would injure the mucosa of the large intestine and decrease the mucosal thickness of the cecum (Yang et al., 2021). A recent report has shown that infiltration of inflammatory cells, ulceration, and epithelial damage were also detected in the cecum and colon of mice fed with HFD (Liu et al., 2021). In addition, gut microbiota mainly in cecum and colon could utilize dietary fiber or other macromolecule, which is not digested by host enzyme, to produce some effective metabolites (Koh et al., 2016). The metabolic disorders induced by HFD may correlate with the injury of cecum, which implied that maintaining cecum function serves as a promising direction for the obesity related diseases. The improved lipid and energy metabolism in liver by Rb1 administration might contribute to the recovery of cecum weight. Allied with the decreased liver weight, Rb1 treatment decreased hepatic triglyceride accumulation and increased Fiaf expression. Although in the majority of the obesity researches, HFD would cause dyslipidemia and therefore increase serum triglyceride level. However, there was no significant difference in serum triglyceride level between NC and HFD group in our result, which was also reported in partial cases (Du et al., 2021; Tang et al., 2021). Gómez-Zorita et al. even observed reduction on the serum triglyceride level in high-fat and high-fructose feeding diet, which might be explained by the decrease in diacylglycerol acyltransferase 2 (Gomez-Zorita et al., 2021). Actually, lowered serum lipid levels are considered as an indicator or common feature in chronic liver disease (Gomez-Zorita et al., 2021). Moreover, PPARs physiological functions involve fatty acid metabolism and sugar metabolism, whose dysregulated mRNA expressions including PPAR*α* and PPAR*γ* in liver, are correlated with triglyceride level in serum (Wu et al., 2021). These overall factors might eventually promote the insignificant changes in serum triglyceride level, which is worthy of further research. Our data showed that Rb1 seemed to have little impact on HFD induced elevated levels of cholesterol and unchanged level of triglyceride in serum, highlighting its potential in the blood glucose regulation.

Given that Rb1 is a representative saponin of ginseng with poor absorption, all of evidences suggested that the effect of Rb1 in glucose-lowering or anti-obesity effect is associated with gut microbiota, which is pivotal in metabolic disease. The results showed that a dramatic shift of gut microbiota was induced in HFD-fed mice by increasing the proportion of *Firmicutes* and decreasing the proportion of *Actinobacteria*, *Tenericutes*, *Bacteroidetes* and *Cyanobacteria*. However, Rb1-treated mice significantly reversed the trend of decreasing *Actinobacteria*. Moreover, relative abundance in *Verrucomicrobia* markedly increased compared to the NC mice (*p* = 0.001013) and HFD group (*p* = 0.001334). Further analysis at the genus level revealed that *Akkermansia* spp.was also significantly elevated compared to the NC and HFD groups. In human, studies have indicated that there is a negative correlation between *Akkermansia* spp. abundance and overweight, obesity, untreated T2DM or hypertension, and *Akkermansia* spp. is strongly related to glucose metabolism and host homeostasis by mediating the negative effects of IFN-*γ* and controlling gut barrier function (Cani, 2018; Wu et al., 2017; Zhang, et al., 2019). Our results showed that Rb1 markedly increased the relative abundance of *Akkermansia* spp. compared with both NC and HFD groups to gain glucose homeostasis.

Previous studies have emphasized the importance of SCFAs such as acetate, propionate, and butyrate in amelioration of chronic inflammatory diseases, promotion of colonocyte health, and metabolic syndrome (Van der Beek, et al., 2017). In current study, the levels of all SCFAs showed reductive tendency in HFD-treated mice, which were consistent with previous report (Tilg and Moschen, 2014). Disappointedly, Rb1 did not reverse this trend, which implied that Rb1 had little impact on the main metabolites of gut microbiota.

The change on the abundance of partial microbiota and little effect on SCFAs intrigued us to further explore the interaction of Rb1 with gut microbiota. PICRUSt was therefore conducted, which suggested that altered microbes were closely related to amino acid metabolism. The results showed that HFD feeding induced a dramatic elevation of Iso, His, Ser, Tyr, Ala and Trp, which is highly consistent with insulin resistance in HFD mice. A recent report highlighted the role of BCAAs especially Iso in the adverse metabolic effects (Yu et al., 2021). In current study, compared to the other two BCAAs, Iso exhibited the best difference between NC and HFD groups, which might recapitulate this point. Rb1 supplementation significantly reduced the levels of Iso, His, Tyr, and Ala in serum. Further studies indicated that Rb1 increased IPA and decreased Trp and IS to maintain Trp homeostasis. The correlation results of other four changed amino acids in HFD mice revealed that alanine was the only amino acid significantly correlated with *Akkermansia* spp.. To the best of our knowledge, this is the first time reported that the change in relative abundance of *Akkermansia* spp. is correlated with circulating level of alanine in serum, which implied that the modulation of alanine might have relationship with *Akkermansia* spp..

## Conclusion

In current work, we investigated the mechanism of anti-diabetic effects of Rb1. The results showed that Rb1 supplementation for mice prevented insulin resistance induced by HFD feeding. Gut microbiota analysis indicated that Rb1 altered the composition of gut flora compared to the HFD-induced mice, especially a higher relative abundance of *Akkermansia* spp.. Further study indicated that Rb1 treatment also maintained the amino acid metabolism homeostasis, especially Leu, Iso, Trp and Ala. In summary, the glucose control mechanism of Rb1 is to alter gut microbiota and modulate amino acid metabolism. Our findings may provide a novel mechanistic understanding of Rb1 in improving insulin sensitivity effects.

## Data Availability

The data presented in the study are deposited in the NCBI repository, accession number PRJNA754322.

## References

[B1] Agus A. Planchais J. Sokol H. (2018). Gut Microbiota Regulation of Tryptophan Metabolism in Health and Disease. Cell Host Microbe 23 (6), 716–724. 10.1016/j.chom.2018.05.003 29902437

[B2] Anhê F. F. Roy D. Pilon G. Dudonné S. Matamoros S. Varin T. V. (2015). A Polyphenol-Rich cranberry Extract Protects from Diet-Induced Obesity, Insulin Resistance and Intestinal Inflammation in Association with Increased *Akkermansia* Spp. Population in the Gut Microbiota of Mice. Gut 64 (6), 872–883. 10.1136/gutjnl-2014-307142 25080446

[B3] Bäckhed F. Ding H. Wang T. Hooper L. V. Koh G. Y. Nagy A. (2004). The Gut Microbiota as an Environmental Factor that Regulates Fat Storage. Proc. Natl. Acad. Sci. U S A. 101 (44), 15718–15723. 10.1073/pnas.0407076101 15505215 PMC524219

[B4] Bai Y. Bao X. Mu Q. Fang X. Zhu R. Liu C. (2021). Ginsenoside Rb1, Salvianolic Acid B and Their Combination Modulate Gut Microbiota and Improve Glucolipid Metabolism in High-Fat Diet Induced Obese Mice. PeerJ 9, e10598. 10.7717/peerj.10598 33604164 PMC7866888

[B5] Bárcena C. Valdés-Mas R. Mayoral P. Garabaya C. Durand S. Rodríguez F. (2019). Healthspan and Lifespan Extension by Fecal Microbiota Transplantation into Progeroid Mice. Nat. Med. 25 (8), 1234–1242. 10.1038/s41591-019-0504-5 31332389

[B6] Berger K. Burleigh S. Lindahl M. Bhattacharya A. Patil P. Stålbrand H. (2021). Xylooligosaccharides Increase *Bifidobacteria* and *Lachnospiraceae* in Mice on a High-Fat Diet, with a Concomitant Increase in Short-Chain Fatty Acids, Especially Butyric Acid. J. Agric. Food Chem. 69, 3617–3625. 10.1021/acs.jafc.0c06279 33724030 PMC8041301

[B7] Canfora E. E. Meex R. C. R. Venema K. Blaak E. E. (2019). Gut Microbial Metabolites in Obesity, NAFLD and T2DM. Nat. Rev. Endocrinol. 15 (5), 261–273. 10.1038/s41574-019-0156-z 30670819

[B8] Cani P. D. (2018). Human Gut Microbiome: Hopes, Threats and Promises. Gut 67 (9), 1716–1725. 10.1136/gutjnl-2018-316723 29934437 PMC6109275

[B9] Chen K. Xie K. Liu Z. Nakasone Y. Sakao K. Hossain A. (2019). Preventive Effects and Mechanisms of Garlic on Dyslipidemia and Gut Microbiome Dysbiosis. Nutrients 11, 1225. 10.3390/nu11061225 PMC6627858 31146458

[B10] Cole J. R. Wang Q. Cardenas E. Fish J. Chai B. Farris R. J. (2008). The Ribosomal Database Project: Improved Alignments and New Tools for rRNA Analysis. Nucleic Acids Res. 37, D141–D145. 10.1093/nar/gkn879 19004872 PMC2686447

[B11] D'Antona G. Ragni M. Cardile A. Tedesco L. Dossena M. Bruttini F. (2010). Branched-chain Amino Acid Supplementation Promotes Survival and Supports Cardiac and Skeletal Muscle Mitochondrial Biogenesis in Middle-Aged Mice. Cell Metab 12 (4), 362–372. 10.1016/j.cmet.2010.08.016 20889128

[B12] Diano S. Horvath T. L. (2012). Mitochondrial Uncoupling Protein 2 (UCP2) in Glucose and Lipid Metabolism. Trends Mol. Med. 18 (1), 52–58. 10.1016/j.molmed.2011.08.003 21917523

[B13] Diez-Echave P. Vezza T. Rodríguez-Nogales A. Hidalgo-Garcia L. Garrido-Mesa J. Ruiz-Malagon A. (2020). The Beneficial Effects of Lippia Citriodora Extract on Diet-Induced Obesity in Mice Are Associated with Modulation in the Gut Microbiota Composition. Mol. Nutr. Food Res. 64, e2000005. 10.1002/mnfr.202000005 32415899

[B14] Du Y. Paglicawan L. Soomro S. Abunofal O. Baig S. Vanarsa K. (2021). Epigallocatechin-3-Gallate Dampens Non-alcoholic Fatty Liver by Modulating Liver Function, Lipid Profile and Macrophage Polarization. Nutrients 13, 599. 10.3390/nu13020599 33670347 PMC7918805

[B15] Edgar R. C. (2013). UPARSE: Highly Accurate OTU Sequences from Microbial Amplicon Reads. Nat. Methods 10 (10), 996–998. 10.1038/nmeth.2604 23955772

[B16] Fan W. Huang Y. Zheng H. Li S. Li Z. Yuan L. (2020). Ginsenosides for the Treatment of Metabolic Syndrome and Cardiovascular Diseases: Pharmacology and Mechanisms. Biomed. Pharmacother. 132, 110915. 10.1016/j.biopha.2020.110915 33254433

[B17] Felig P. (1973). The Glucose-Alanine Cycle. Metabolism 22 (2), 179–207. 10.1016/0026-0495(73)90269-2 4567003

[B18] Foretz M. Guigas B. Viollet B. (2019). Understanding the Glucoregulatory Mechanisms of Metformin in Type 2 Diabetes Mellitus. Nat. Rev. Endocrinol. 15 (10), 569–589. 10.1016/0026-0495(73)90269-210.1038/s41574-019-0242-2 31439934

[B19] Francque S. Szabo G. Abdelmalek M. F. Byrne C. D. Cusi K. Dufour J. F. (2021). Nonalcoholic Steatohepatitis: the Role of Peroxisome Proliferator-Activated Receptors. Nat. Rev. Gastroenterol. Hepatol. 18 (1), 24–39. 10.1038/s41575-020-00366-5 33093663

[B20] Gómez-Zorita S. Milton-Laskibar I. Macarulla M. T. Biasutto L. Fernández-Quintela A. Miranda J. (2021). Pterostilbene Modifies Triglyceride Metabolism in Hepatic Steatosis Induced by High-Fat High-Fructose Feeding: a Comparison with its Analog Resveratrol. Food Funct. 12, 3266–3279. 10.1039/d0fo03320k 33877249

[B21] Hasanpour M. Iranshahy M. Iranshahi M. (2020). The Application of Metabolomics in Investigating Anti-diabetic Activity of Medicinal Plants. Biomed. Pharmacother. 128, 110263. 10.1016/j.biopha.2020.110263 32450525

[B22] Holeček M. (2020). Why Are Branched-Chain Amino Acids Increased in Starvation and Diabetes? Nutrients 12, 3087. 10.3390/nu12103087 PMC7600358 33050579

[B23] Huang Y. Zhou J. Wang S. Xiong J. Chen Y. Liu Y. (2020). Indoxyl Sulfate Induces Intestinal Barrier Injury through IRF1-DRP1 axis-mediated Mitophagy Impairment. Theranostics 10 (16), 7384–7400. 10.7150/thno.45455 32641998 PMC7330852

[B24] Jami E. Israel A. Kotser A. Mizrahi I. (2013). Exploring the Bovine Rumen Bacterial Community from Birth to Adulthood. ISME J. 7 (6), 1069–1079. 10.1038/ismej.2013.2 23426008 PMC3660679

[B25] Jiang X. T. Peng X. Deng G. H. Sheng H. F. Wang Y. Zhou H. W. (2013). Illumina Sequencing of 16S rRNA Tag Revealed Spatial Variations of Bacterial Communities in a Mangrove Wetland. Microb. Ecol. 66 (1), 96–104. 10.1007/s00248-013-0238-8 23649297

[B26] Karusheva Y. Koessler T. Strassburger K. Markgraf D. Mastrototaro L. Jelenik T. (2019). Short-term Dietary Reduction of Branched-Chain Amino Acids Reduces Meal-Induced Insulin Secretion and Modifies Microbiome Composition in Type 2 Diabetes: a Randomized Controlled Crossover Trial. Am. J. Clin. Nutr. 110 (5), 1098–1107. 10.1093/ajcn/nqz191 31667519 PMC6821637

[B27] Kim J. D. Yoon N. A. Jin S. Diano S. (2019). Microglial UCP2 Mediates Inflammation and Obesity Induced by High-Fat Feeding. Cell Metab. 30 (5), 952–e5. 10.1016/j.cmet.2019.08.010 31495690 PMC7251564

[B28] Koh A. De Vadder F. Kovatcheva-Datchary P. Bäckhed F. (2016). From Dietary Fiber to Host Physiology: Short-Chain Fatty Acids as Key Bacterial Metabolites. Cell 165, 1332–1345. 10.1016/j.cell.2016.05.041 27259147

[B29] Kusminski C. M. Bickel P. E. Scherer P. E. (2016). Targeting Adipose Tissue in the Treatment of Obesity-Associated Diabetes. Nat. Rev. Drug Discov. 15 (9), 639–660. 10.1038/nrd.2016.75 27256476

[B30] Langille M. G. Zaneveld J. Caporaso J. G. McDonald D. Knights D. Reyes J. A. (2013). Predictive Functional Profiling of Microbial Communities Using 16S rRNA Marker Gene Sequences. Nat. Biotechnol. 31 (9), 814–821. 10.1038/nbt.2676 23975157 PMC3819121

[B31] Ley R. E. Bäckhed F. Turnbaugh P. Lozupone C. A. Knight R. D. Gordon J. I. (2005). Obesity Alters Gut Microbial Ecology. Proc. Natl. Acad. Sci. U S A. 102 (31), 11070–11075. 10.1073/pnas.0504978102 16033867 PMC1176910

[B32] Li J. Li R. Li N. Zheng F. Dai Y. Ge Y. (2018). Mechanism of Antidiabetic and Synergistic Effects of Ginseng Polysaccharide and Ginsenoside Rb1 on Diabetic Rat Model. J. Pharm. Biomed. Anal. 158, 451–460. 10.1016/j.jpba.2018.06.024 30032757

[B33] Liu D. Ji Y. Guo Y. Wang H. Wu Z. Li H. (2021). Dietary Supplementation of Apple Phlorizin Attenuates the Redox State Related to Gut Microbiota Homeostasis in C57BL/6J Mice Fed with a High-Fat Diet. J. Agric. Food Chem. 69, 198–211. 10.1021/acs.jafc.0c06426 33350821

[B34] Lynch C. J. Adams S. H. (2014). Branched-chain Amino Acids in Metabolic Signalling and Insulin Resistance. Nat. Rev. Endocrinol. 10 (12), 723–736. 10.1038/nrendo.2014.171 25287287 PMC4424797

[B35] Massafra V. Milona A. Vos H. R. Ramos R. J. J. Gerrits J. Willemsen E. C. L. (2017). Farnesoid X Receptor Activation Promotes Hepatic Amino Acid Catabolism and Ammonium Clearance in Mice. Gastroenterology 152 (6), 1462–e10. 10.1053/j.gastro.2017.01.014 28130067

[B36] Meijnikman A. S. Gerdes V. E. Nieuwdorp M. Herrema H. (2018). Evaluating Causality of Gut Microbiota in Obesity and Diabetes in Humans. Endocr. Rev. 39 (2), 133–153. 10.1210/er.2017-00192 29309555

[B37] Nahin R. L. Barnes P. M. Stussman B. J. Bloom B. (2009). Costs of Complementary and Alternative Medicine (CAM) and Frequency of Visits to CAM Practitioners: United States 2007. Natl. Health Stat. Rep. 30 (18):1-14. 19771719

[B38] Newgard C. B. (2012). Interplay between Lipids and Branched-Chain Amino Acids in Development of Insulin Resistance. Cell Metab. 15 (5), 606–614. 10.1016/j.cmet.2012.01.024 22560213 PMC3695706

[B39] Nie Q. Chen H. Hu J. Gao H. Fan L. Long Z. (2018). Arabinoxylan Attenuates Type 2 Diabetes by Improvement of Carbohydrate, Lipid, and Amino Acid Metabolism. Mol. Nutr. Food Res. 62 (20), e1800222. 10.1002/mnfr.201800222 30211972

[B40] Pawlak M. Lefebvre P. Staels B. (2015). Molecular Mechanism of PPARα Action and its Impact on Lipid Metabolism, Inflammation and Fibrosis in Non-alcoholic Fatty Liver Disease. J. Hepatol. 62 (3), 720–733. 10.1016/j.jhep.2014.10.039 25450203

[B41] Petersen M. C. Vatner D. F. Shulman G. I. (2017). Regulation of Hepatic Glucose Metabolism in Health and Disease. Nat. Rev. Endocrinol. 13 (10), 572–587. 10.1038/nrendo.2017.80 28731034 PMC5777172

[B42] Plovier H. Everard A. Druart C. Depommier C. Van Hul M. Geurts L. (2017). A Purified Membrane Protein from *Akkermansia Muciniphila* or the Pasteurized Bacterium Improves Metabolism in Obese and Diabetic Mice. Nat. Med. 23 (1), 107–113. 10.1038/nm.4236 27892954

[B43] Sánchez B. Delgado S. Blanco-Míguez A. Lourenço A. Gueimonde M. Margolles A. (2017). Probiotics, Gut Microbiota, and Their Influence on Host Health and Disease. Mol. Nutr. Food Res. 61, 1600240. 10.1002/mnfr.201600240 27500859

[B44] Shen H. Leung W. I. Ruan J. Q. Li S. L. Lei J. P. Wang Y. T. (2013). Biotransformation of Ginsenoside Rb1 via the Gypenoside Pathway by Human Gut Bacteria. Chin. Med. 8 (1), 22. 10.1186/1749-8546-8-22 24267405 PMC4175505

[B45] Shergis J. L. Zhang A. L. Zhou W. Xue C. C. (2013). Panax Ginseng in Randomised Controlled Trials: a Systematic Review. Phytother. Res. 27 (7), 949–965. 10.1002/ptr.4832 22969004

[B46] Tang S. Shi Z. Qiao X. Zhuang Z. Ding Y. Wu Y. (2021). Carya Cathayensis Leaf Extract Attenuates Ectopic Fat Deposition in Liver, Abdomen and Aortic Arch in Ovariectomized Rats Fed a High-Fat Diet. Phytomedicine 82, 153447. 10.1016/j.phymed.2020.153447 33444943

[B47] Tilg H. Moschen A. R. (2014). Microbiota and Diabetes: an Evolving Relationship. Gut 63 (9), 1513–1521. 10.1136/gutjnl-2014-306928 24833634

[B48] Turnbaugh P. J. Hamady M. Yatsunenko T. Cantarel B. L. Duncan A. Ley R. E. (2009). A Core Gut Microbiome in Obese and Lean Twins. Nature 457 (7228), 480–484. 10.1038/nature07540 19043404 PMC2677729

[B49] Van der Beek C. M. Dejong C. H. C. Troost F. J. Masclee A. A. M. Lenaerts K. (2017). Role of Short-Chain Fatty Acids in Colonic Inflammation, Carcinogenesis, and Mucosal protection and Healing. Nutr. Rev. 75 (4), 286–305. 10.1093/nutrit/nuw067 28402523

[B50] Venkatesh M. Mukherjee S. Wang H. Li H. Sun K. Benechet A. P. (2014). Symbiotic Bacterial Metabolites Regulate Gastrointestinal Barrier Function via the Xenobiotic Sensor PXR and Toll-like Receptor 4. Immunity 41 (2), 296–310. 10.1016/j.immuni.2014.06.014 25065623 PMC4142105

[B51] Wang Y. Sheng H. F. He Y. Wu J. Y. Jiang Y. X. Tam N. F. (2012). Comparison of the Levels of Bacterial Diversity in Freshwater, Intertidal Wetland, and Marine Sediments by Using Millions of Illumina Tags. Appl. Environ. Microbiol. 78 (23), 8264–8271. 10.1128/AEM.01821-12 23001654 PMC3497375

[B52] Watanabe M. Houten S. M. Mataki C. Christoffolete M. A. Kim B. W. Sato H. (2006). Bile Acids Induce Energy Expenditure by Promoting Intracellular Thyroid Hormone Activation. Nature 439 (7075), 484–489. 10.1038/nature04330 16400329

[B53] Wong A. S. Che C. M. Leung K. W. (2015). Recent Advances in Ginseng as Cancer Therapeutics: a Functional and Mechanistic Overview. Nat. Prod. Rep. 32 (2), 256–272. 10.1039/c4np00080c 25347695

[B54] Wu W. Lv L. Shi D. Ye J. Fang D. Guo F. (2017). Protective Effect of *Akkermansia Muciniphila* against Immune-Mediated Liver Injury in a Mouse Model. Front. Microbiol. 8, 1804. 10.3389/fmicb.2017.01804 29033903 PMC5626943

[B55] Wu Y. Sun H. Yi R. Tan F. Zhao X. (2021). Anti‐obesity Effect of Liupao tea Extract by Modulating Lipid Metabolism and Oxidative Stress in High‐fat‐diet‐induced Obese Mice. J. Food Sci. 86, 215–227. 10.1111/1750-3841.15551 33300164

[B56] Yang Q. Vijayakumar A. Kahn B. B. (2018). Metabolites as Regulators of Insulin Sensitivity and Metabolism. Nat. Rev. Mol. Cell Biol. 19 (10), 654–672. 10.1038/s41580-018-0044-8 30104701 PMC6380503

[B57] Yang X. Y. Zhong D. Y. Wang G. L. Zhang R. G. Zhang Y. L. (2021). Effect of Walnut Meal Peptides on Hyperlipidemia and Hepatic Lipid Metabolism in Rats Fed a High-Fat Diet. Nutrients 13, 1410. 10.3390/nu13051410 33922242 PMC8146006

[B58] Yoon S. J. Kim S. K. Lee N. Y. Choi Y. R. Kim H. S. Gupta H. (2021). Effect of Korean Red Ginseng on Metabolic Syndrome. J. Ginseng Res. 45 (3), 380–389. 10.1016/j.jgr.2020.11.002 34025131 PMC8134847

[B59] Yu D. Richardson N. E. Green C. L. Spicer A. B. Murphy M. E. Flores V. (2021). The Adverse Metabolic Effects of Branched-Chain Amino Acids Are Mediated by Isoleucine and Valine. Cell Metab. 33 (5), 905–e6. 10.1016/j.cmet.2021.03.025 33887198 PMC8102360

[B60] Zeng S. L. Li S. Z. Xiao P. T. Cai Y. Y. Chu C. Chen B. Z. (2020). Citrus Polymethoxyflavones Attenuate Metabolic Syndrome by Regulating Gut Microbiome and Amino Acid Metabolism. Sci. Adv. 6 (1), eaax6208. 10.1126/sciadv.aax6208 31922003 PMC6941918

[B61] Zhang T. Li Q. Cheng L. Buch H. Zhang F. (2019). *Akkermansia Muciniphila* Is a Promising Probiotic. Microb. Biotechnol. 12 (6), 1109–1125. 10.1111/1751-7915.13410 31006995 PMC6801136

[B62] Zhou P. Xie W. He S. Sun Y. Meng X. Sun G. (2019). Ginsenoside Rb1 as an Anti-diabetic Agent and its Underlying Mechanism Analysis. Cells 8, 204. 10.3390/cells8030204 PMC6468558 30823412

