# Supplementary material for: Ginsenoside Rb1 ameliorates Glycemic Disorder in Mice With High Fat Diet-Induced Obesity via Regulating Gut Microbiota and Amino Acid Metabolism
Source: Front Pharmacol. 2021 Nov 24;12:756491. doi: 10.3389/fphar.2021.756491 (PMC8654325; doi:10.3389/fphar.2021.756491)
Supplement: Supplementary file 1 [file DataSheet1.docx]

**Ginsenoside Rb1 ameliorates Glycemic Disorder in Mice with High Fat Diet-induced obesity *via* Regulating Gut Microbiota and Amino Acid Metabolism**

**Table S1** Diet composition

| Compositions | Normal diet | | High fat diet | |
| --- | --- | --- | --- | --- |
|  | g (%) | kcal (%) | g (%) | kcal (%) |
| protein | 20 | 23.85 | 15.24 | 14.85 |
| fat | 4 | 11.16 | 18.06 | 39.59 |
| fiber | 5 | 0 | 1.67 | 0 |
| moisture | 10 | 0 | 11.41 | 0 |
| ash content | 8 | 0 | 4.28 | 0 |
| Ca | 1 | 0 | 0.73 | 0 |
| P | 0.6 | 0 | 0.45 | 0 |
| carbohydrate | 55.03 | 64.99 | 46.76 | 46.57 |
| Energy (kcal/g diet) | 3.39 | | 4.1 | |

**Supplementary Figure 1.** ^1^H NMR spectrum for ginsenoside Rb1.

**Supplementary Figure 2.** ^13^C NMR spectrum for ginsenoside Rb1.


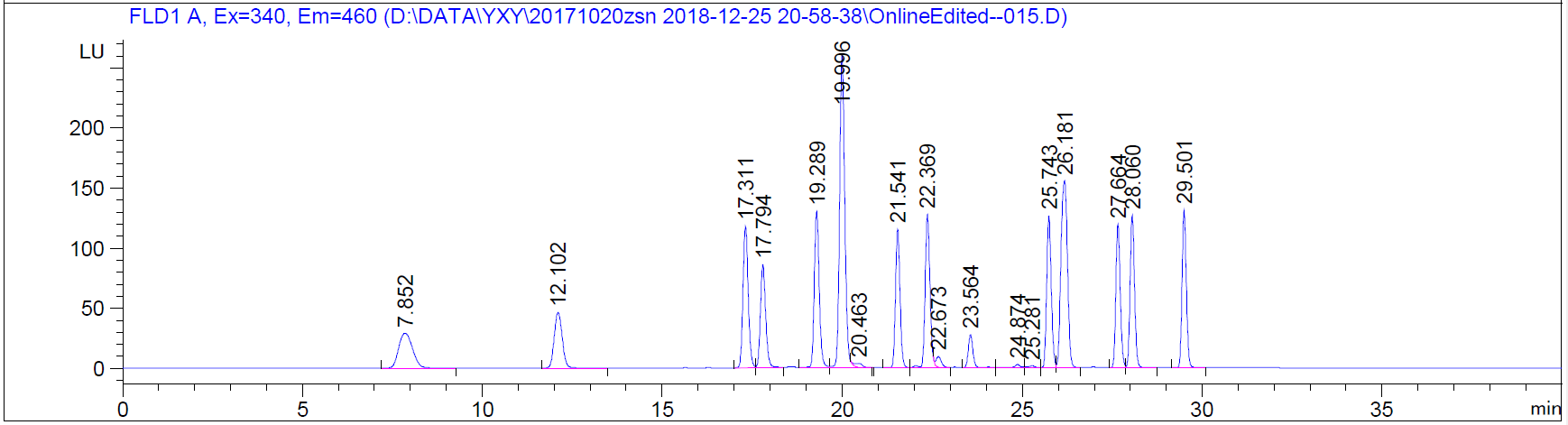


**Supplementary Figure 3.** BCAA spectrum for amino acids mix standard.

**
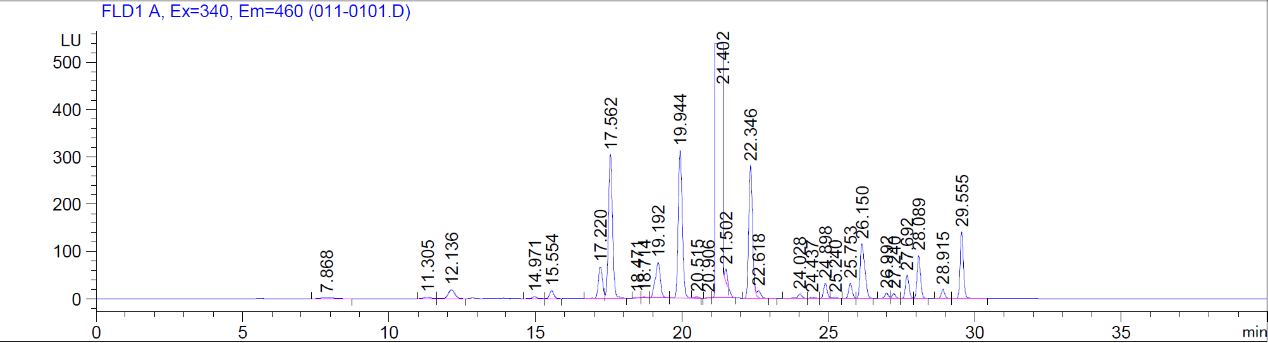
**

**Supplementary Figure 4.** BCAA spectrum for sample from samples.


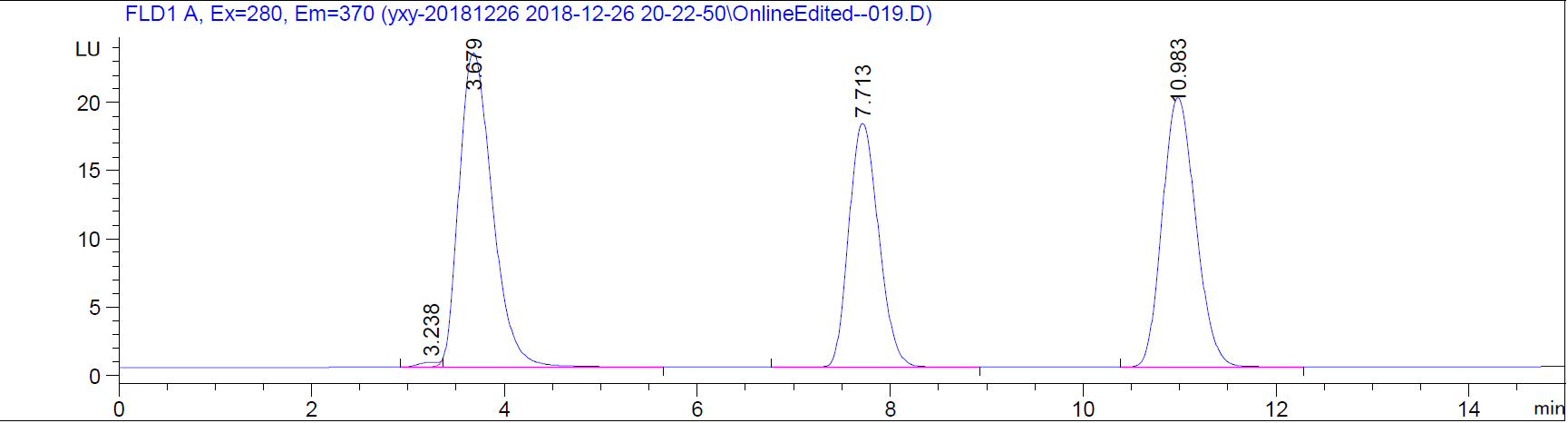


**Supplementary Figure 5.** IPA spectrum for indoxyl sulfate (IS), indole-3-acetic acid (IAA) and 3-Indolepropionic acid (IPA) standard.


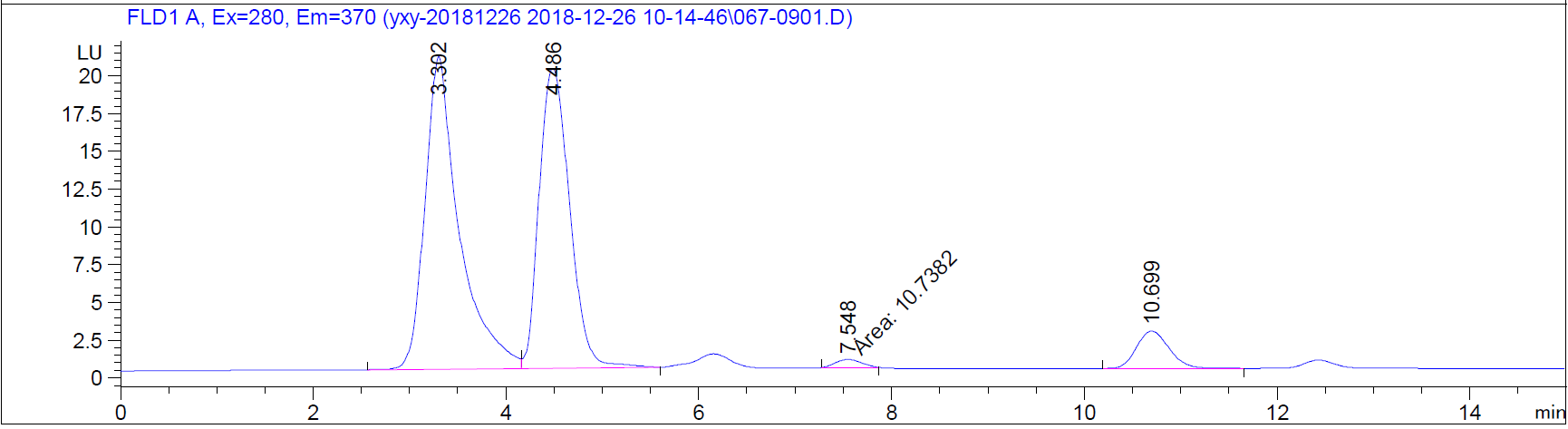


**Supplementary Figure 6.** IPA spectrum for sample from normal chow-diet group.

**Supplementary Figure 7.** (A) Total cholesterol in serum. (B) Triglycerides in serum.

**Supplementary Figure 8.** Refraction curves and Shannon-Winner curves. (A) Refraction curve; (B) Shannon-Winner curves.

**Supplementary Figure 9.** Gut microbiota analysis of relative abundance in phylum (A), order (B), and genus (C) level.

**Supplementary Figure 10.** The effects of Rb1 supplementation on SCFAs metabolism. Mice were fed a normal chow (Control) diet or high-fat-diet (HFD) for 12 weeks. HFD-fed animals were treated with a daily dose of Rb1 (200 mg/kg). (A) Acetic acid. (B) Propionic acid. (C) Butyrate. (D) Isobutyric acid. (E) Valeric acid. (F) Isovaleric acid. The data are expressed as the mean ± SEM; n = 6; one-way analysis of variance with Tukey’s post-hoc test. ^#^*P*<0.05, ^# #^ *P*<0.01, for NC vs HFD; ^*^*P*<0.05, ^**^*P*<0.01 for Rb1 vs HFD.
